# Thyroid tuberculosis mimicking multinodular goiter: a case report

**DOI:** 10.1186/s13256-024-04592-2

**Published:** 2024-07-09

**Authors:** Endeshaw Asaye Kindie, Tigist Hailu Belachew, Lidetu Temeche Habte, Samuel Addisu Abera, Addisu Minaye Dejen, Sileshi Ayele Abebe, Yohannis Derbew Molla

**Affiliations:** 1 Department of Pathology, School of Medicine, University of Gondar College of Medicine and Health Sciences, Gondar, Ethiopia; 2 Department of Radiology, School of Medicine, University of Gondar College of Medicine and Health Sciences, Gondar, Ethiopia; 3 Department of Gynecology and Obstetrics, School of Medicine, University of Gondar College of Medicine and Health Sciences, Gondar, Ethiopia; 4 Department of Surgery, School of Medicine, University of Gondar College of Medicine and Health Sciences, Gondar, Ethiopia

**Keywords:** Tuberculosis, Thyroid, Anti-tuberculosis, Abscess

## Abstract

**Background:**

*Mycobacterium tuberculosis* is the second most common infectious cause of death in adults worldwide. The ability of this organism to efficiently establish latent infection has enabled it to spread to nearly one-third of individuals worldwide. Approximately 8 million new cases of active tuberculosis disease occur each year, leading to about 1.7 million deaths. The disease incidence is magnified by the concurrent epidemic of human immunodeficiency virus infection. A total of 1.3 million people died from tuberculosis in 2022. In 2022, an estimated 10.6 million people fell ill with tuberculosis worldwide, including 5.8 million men, 3.5 million women, and 1.3 million children. We report a case of thyroid tuberculosis presenting as multinodular goiter. Neck ultrasound was done and revealed abscess collection on the background of multinodular colloid goiter. The diagnosis of thyroid tuberculosis was confirmed by a positive GeneXpert of the pus sample and the presence of extensive caseous necrosis on cytopathology examination. Furthermore, anterior neck swelling may provide a diagnostic challenge by clinically mimicking multinodular goiter or thyroid neoplasms. Owing to its rarity and its tendency to pose a clinical diagnostic challenge, we decided to report it.

**Case presentation:**

A 60-year-old retired female Ethiopian high-school teacher presented to University of Gondar Hospital, Gondar, Ethiopia with firm, nontender multinodular anterior neck swelling measuring at largest 2 × 3 cm that moves with swallowing. GeneXpert of the pus sample and cytopathology examination confirmed the diagnosis of thyroid tuberculosis, and the patient was started on 2 rifampicin−ethambutol−isoniazid−pyrazinamide/4 rifampicin−isoniazid 3 tablets by mouth/day, which is defined as the preferred first-line anti-tuberculosis regimen in Ethiopia, and pyridoxine 50 mg by mouth per day for 6 months. Since then, she has been followed with regular liver function tests. The patient has shown a smooth course with no significant adverse effects encountered. Currently, the patient has completed her anti-tuberculosis treatment and is doing well.

**Conclusion:**

In the clinical evaluation of a patient with anterior neck swelling, tuberculosis must be considered as a differential diagnosis in subjects from endemic areas for early diagnostic workup and management.

## Background

Tuberculosis is caused by the bacterium *Mycobacterium tuberculosis*. The bacteria usually attack the lungs, but TB bacteria can attack any part of the body such as the kidney, spine, and brain. TB bacteria spread through the air from one person to another. When a person with TB disease of the lungs or throat coughs, speaks, or sings, TB bacteria can get into the air. People nearby may breathe in these bacteria and become infected. Not everyone infected with TB bacteria becomes sick. As a result, two TB-related conditions exist: latent TB infection and TB disease. TB bacteria can live in the body without making you sick. This is called latent TB infection. In most people who breathe in TB bacteria and become infected, the body is able to fight the bacteria to stop them from growing. People with latent TB infection have no symptoms, do not feel sick, cannot spread TB bacteria to others, usually having a positive TB skin test reaction or positive TB blood test, but may develop TB disease if they do not receive treatment for latent TB infection. Many people who have latent TB infection never develop TB disease. In these people, the TB bacteria remain inactive for their lifetime without causing disease. However, in other people, especially those with a weak immune system, the bacteria become active, multiply, and cause TB disease. TB bacteria become active if the immune system cannot stop them from growing. When TB bacteria are active (multiplying in your body), this is called TB disease. People with TB disease are sick. They may also be able to spread the bacteria to people they spend time with every day. Some people develop TB disease soon after becoming infected (within weeks) before their immune system can fight the TB bacteria. Other people may get sick years later when their immune system becomes weak for another reason. For people whose immune systems are weak, especially those with HIV infection, the risk of developing TB disease is much higher than for people with normal immune systems. Symptoms of TB disease depend on where in the body the TB bacteria are growing. TB bacteria usually grow in the lungs (pulmonary TB). TB disease in the lungs may cause symptoms such as a bad cough that lasts 3 weeks or longer, pain in the chest, and coughing up blood or sputum (phlegm from deep inside the lungs). Other symptoms of TB disease are weakness or fatigue, weight loss, no appetite, chills, and fever and sweating at night. Symptoms of TB disease in other parts of the body depend on the area affected. Generally, persons at high risk for developing TB disease fall into two categories: persons who have been recently infected with TB bacteria and persons with medical conditions that weaken the immune system. Persons who have been recently infected with TB bacteria include close contacts of a person with infectious TB disease, persons who have immigrated from areas of the world with high rates of TB, children less than 5 years of age who have a positive TB test, groups with high rates of TB transmission such as homeless persons, injection drug users, persons with HIV infection, and persons who work or reside with people who are at high risk for TB in facilities or institutions such as hospitals, homeless shelters, correctional facilities, nursing homes, and residential homes for those with HIV [1].

Persons with medical conditions that weaken the immune system include babies and young children, and people with any of the following conditions: HIV infection (the virus that causes AIDS), substance abuse, silicosis, diabetes mellitus, severe kidney disease, low body weight, head and neck cancer, medical treatments such as corticosteroids or organ transplant, and specialized treatment for rheumatoid arthritis or Crohn’s disease [1].

In Ethiopia, TB is still a major public health problem. The country is still among the 22 high-TB-burden countries with high number of missed and infectious TB cases in the community [2], with an annual TB incidence of 119 cases per 100,000 population in 2021 [3]. The overall pooled prevalence of TB in Ethiopia was 0.19% [95% confidence interval (CI): 0.12–0.28%] [4]. An estimated global total of 10.6 million people (95% uncertainty interval [UI]: 9.9–11.4 million) fell ill with TB in 2022, equivalent to 133 incident cases (95% UI: 124–143) per 100 000 population. Among all incident TB cases, 6.3% were among people living with HIV. Most TB cases in 2022 were in the WHO regions of South-East Asia (46%), Africa (23%), and the Western Pacific (18%), with smaller shares in the Eastern Mediterranean (8.1%), the Americas (3.1%), and Europe (2.2%) [5]. Thyroid tuberculosis is a rare disease. Its incidence is low even in countries where prevalence of pulmonary tuberculosis is high (0.1–0.4%). In literature, there are only a few cases which were diagnosed as thyroid tuberculosis. It can be explained by a high resistance of the thyroid gland to infectious processes. However, the prevalence of tuberculosis has increased worldwide, and thyroid involvement can be a primary manifestation of the disease. The most frequent clinical presentation is a solitary thyroid nodule that may present as a cystic nodule. It may also present as thyroid abscess with pain, fever, and other nonspecific signs and symptoms [6]. Primary thyroid tuberculosis is rare even in countries where TB disease is endemic, with prevalence ranging from 0.1% to 1.15% [7]. Some risk factors such as age, diabetes mellitus, malnutrition, and acquired immunodeficiency syndrome were associated with the occurrence of tuberculosis of the thyroid gland [8, 9]. Our patient was at risk for developing tuberculosis as she was from an endemic area and was 60 years old.

We report herein a case of thyroid tuberculosis presenting as anterior neck swelling that was clinically considered as multinodular colloid goiter. Additionally, as evident from our case presentation, the condition can pose a diagnostic challenge for clinicians by resembling multinodular goiter or thyroid neoplasms. As a result, we report this case owing to its rarity and emphasize the importance of considering thyroid tuberculosis in the differential diagnosis of anterior neck swelling for early diagnostic workup and management.

## Case presentation

**Fig. 1:**
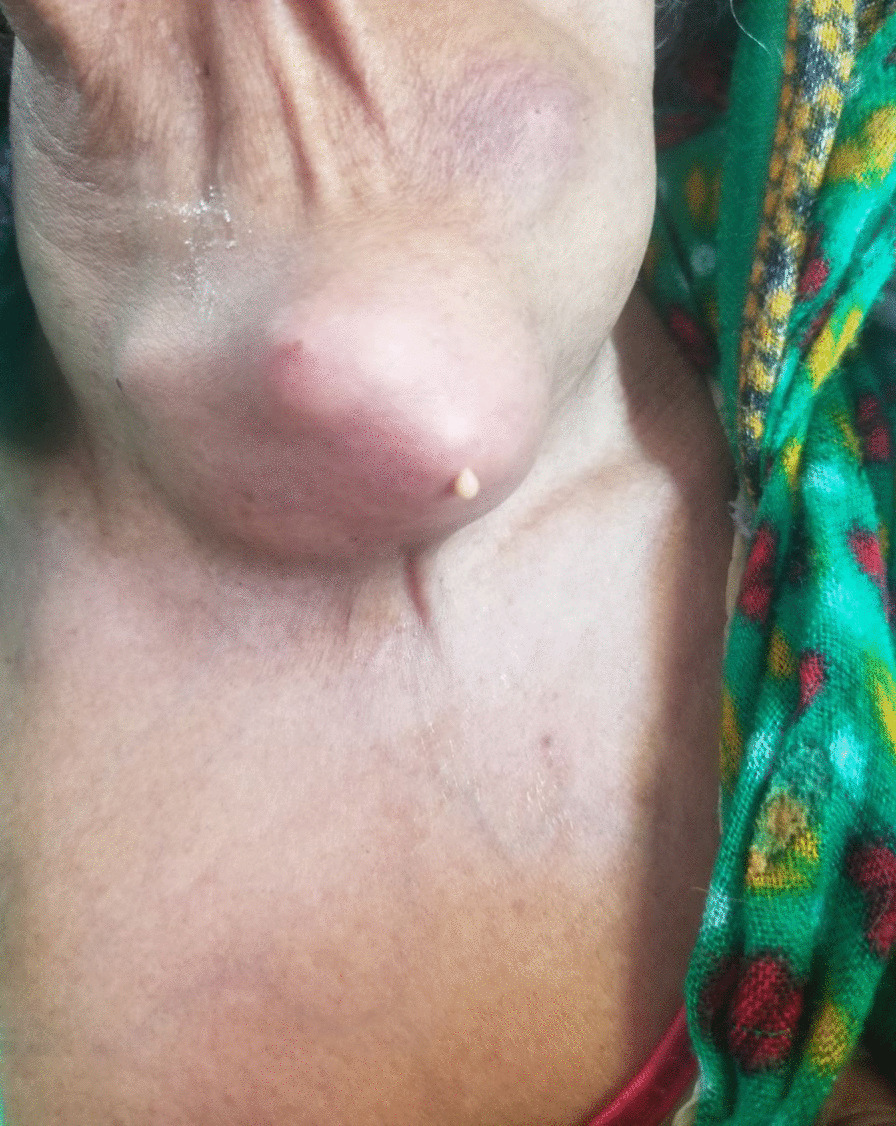
A 60-year-old Ethiopian woman presented with thyroid tuberculosis with postprocedural pus discharge

**Fig. 2:**
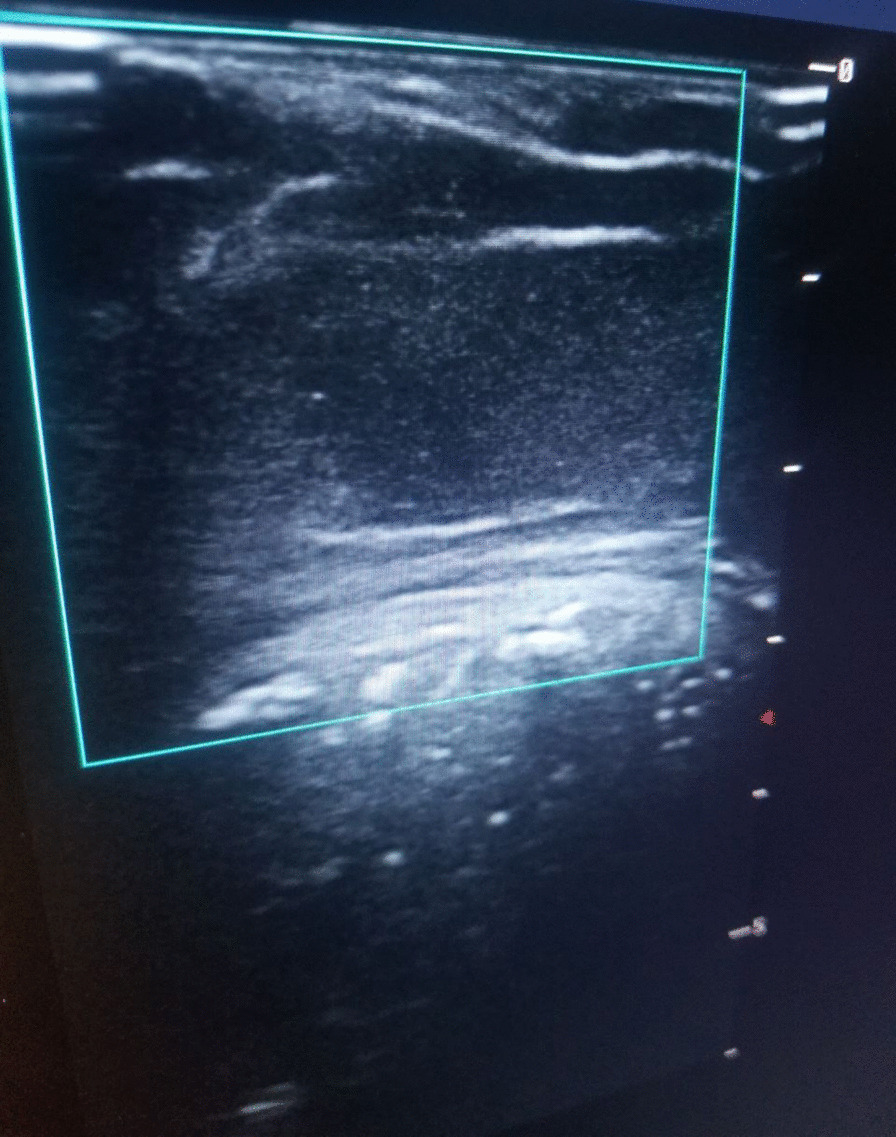
Neck ultrasound of thyroid tuberculosis revealing abscess collections on the left lobe of thyroid on the background of multinodular goiter

**Fig. 3:**
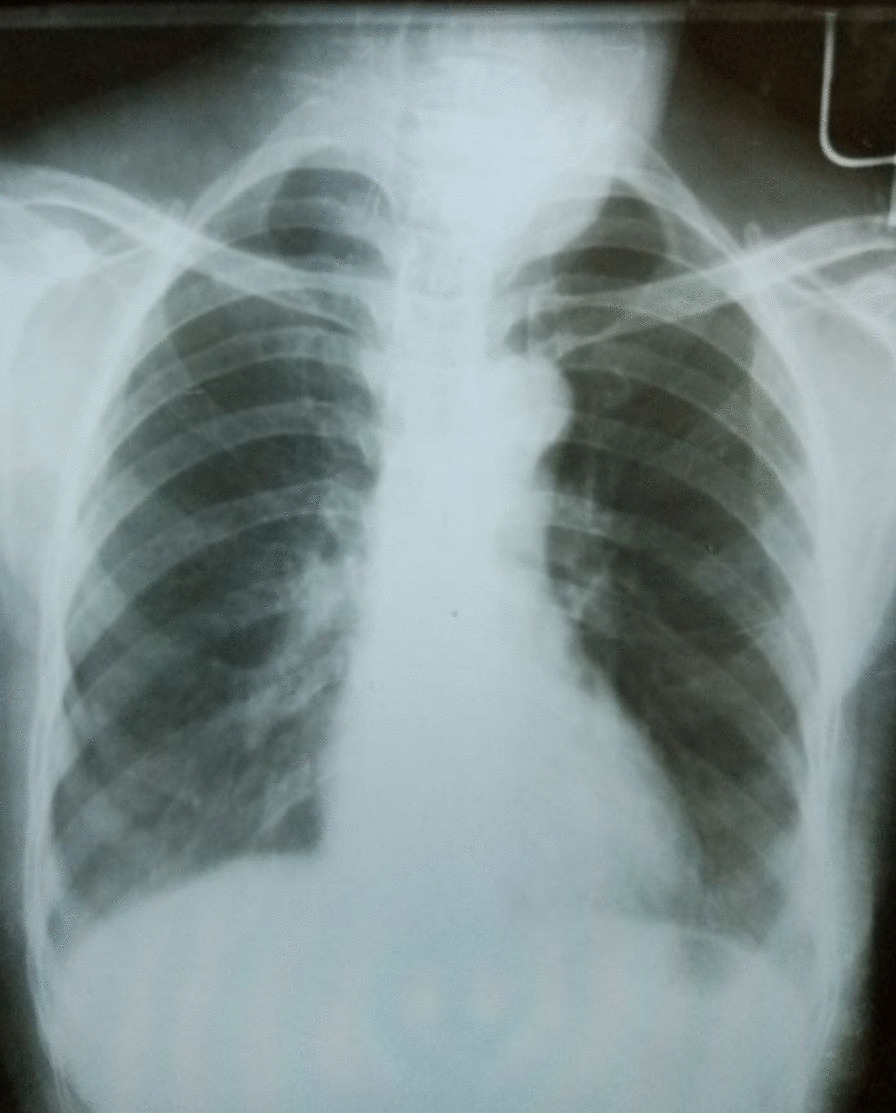
Chest X-ray revealing normal-looking lung parenchyma and thyroid mass

**Fig. 4:**
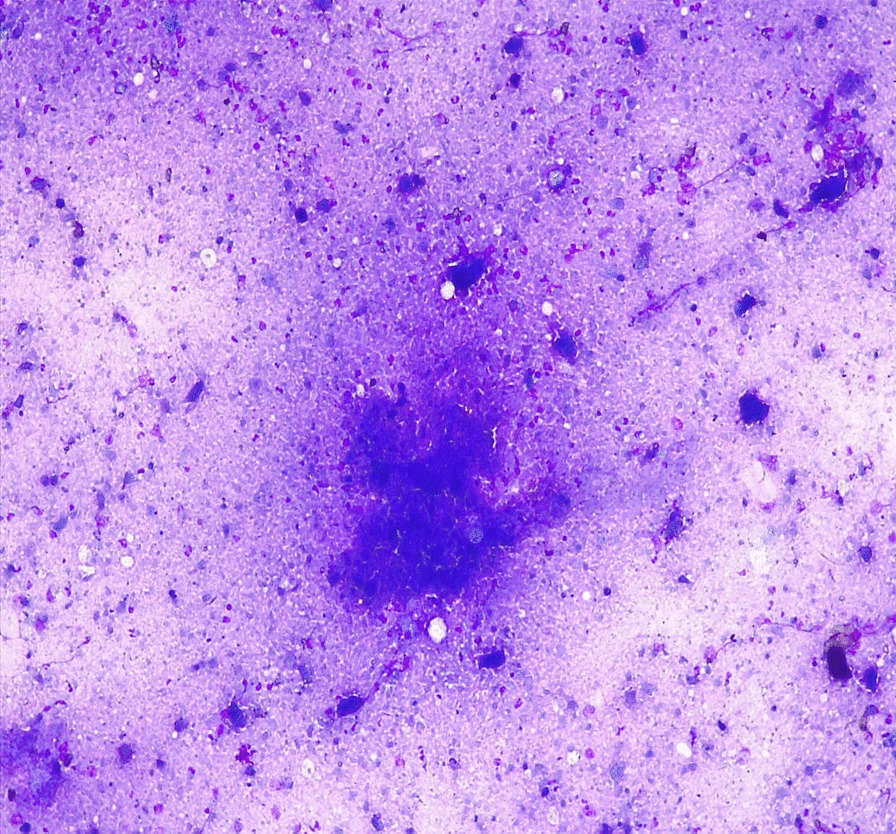
Fine-needle aspiration cytology smear of thyroid tuberculosis showing extensive caseous necrosis on a dirty background (Wright’s stain)

A-60-year-old retired female Ethiopian high-school teacher presented to University of Gondar College of Medicine and Health Sciences, Gondar, Ethiopia with a complaint of anterior neck swelling of 6 years duration with recent increment in size 3 months prior to her current presentation. She had visited a nearby health center for this complaint and took unspecified antibiotics, but with no improvement. She had history of low-grade intermittent fever, unquantified weight loss, and night sweating but with no history of cough, loss of appetite, pressure effects, or bowel, bladder, joint, or nervous system involvement. She had no family history (first-degree relatives) of diabetes, hypertension, or any other remarkable noncommunicable disease, including cancer. Her past medical history is not significant. She had no history of admission to hospital. She had no history of any form of surgical procedures. On physical examination, there was a soft to firm multinodular anterior neck mass largest measuring 2 × 3 cm on the left lobe of thyroid gland (Fig. 1). She did not have axillary, cervical, or intraabdominal lymphadenopathy. On the basis of the above findings, a provisional clinical impression of multinodular goiter ruling out follicular neoplasm was considered. Liver was not palpable below costal margin. There was no splenomegaly. Other clinical findings were within normal limits. Laboratory investigations done on the same day of her presentation, including complete blood count (CBC), erythrocyte sedimentation rate (ESR), and electrocardiogram (ECG), were noncontributory. On CBC, total white blood cell (WBC) count was 4000 µL with 54% granulocytes, 42% lymphocytes, 2% eosinophils, and 2% monocytes. Platelet count was 350,000 µL. Hemoglobin was 14.5 g/dL with mean corpuscular volume (MCV) of 85 fL. ESR was 75 mm/hour. GeneXpert for detection of *Mycobacterium tuberculosis* in pus samples was positive. Renal function test revealed blood urea nitrogen (BUN) of 14 mg/dL and serum creatinine level was 0.7 mg/dL. On liver function test, total bilirubin was 0.6 mg/dL, serum albumin was 4.2 g/dL, and serum aspartate transaminase (AST/SGOT) and serum alanine transaminase (ALT/SGPT) were 32 and 34 IU/L, respectively. Urinalysis was also done and was normal. She was screened for RVI and found to be nonreactive. Serum TSH, total T4, and total T3 values were 3 µIU/mL, 7 µg/dL, and 1.5 ng/mL, respectively. Neck ultrasound was done and revealed abscess collections on the left lobe of thyroid on the background of multinodular goiter (Fig. 2). Chest X-ray was done and revealed normal-looking lung parenchyma and thyroid mass (Fig. 3). Abdominopelvic ultrasound was done and revealed normal study. Owing to a limited number of pathologists and a long waiting list of patients, she underwent fine-needle aspiration cytology (FNAC) after 2 weeks from initial presentation. Repeated FNAC smears showed extensive caseous necrosis on a dirty background (Fig. 4).

With the above cytopathology finding and positive GeneXpert of the pus sample, the diagnosis of thyroid tuberculosis was rendered and the patient was started on 2 RHZE/4 RH 3 tablets by mouth per day, which is defined as the preferred first-line anti-TB regimen in Ethiopia, and pyridoxine 50 mg by mouth/day for 6 months. Thereafter, she was followed with regular liver function tests. The patient has had a smooth course with no significant adverse effects encountered. Currently, the patient has completed her anti-TB treatment and is doing well. After completion of her anti-TB treatment, the patient had neck ultrasound examination that revealed multinodular goiter with no abscess collection.

## Discussion

Tuberculosis is a communicable disease that is a major cause of ill health and one of the leading causes of death worldwide. Until the coronavirus (COVID-19) pandemic, TB was the leading cause of death from a single infectious agent, ranking above HIV/AIDS. Estimating TB disease burden during the COVID-19 pandemic is difficult and relies heavily on country- and region-specific dynamic models for low- and middle-income countries. New national population-based surveys of TB disease and up-to-date cause-of-death data from national vital registration systems of high quality and coverage are needed for more accurate estimation in the wake of the pandemic [10]. Tuberculosis can involve virtually any organ or tissue in the body. Nonpulmonary sites tend to be more common among children and persons with impaired immunity. To establish the diagnosis of extrapulmonary tuberculosis, appropriate specimens including pleural fluid; pericardial or peritoneal fluid; pleural, pericardial, and peritoneal biopsy specimens; lymph node tissue; and bone marrow, bone, blood, urine, brain, or cerebrospinal fluid should be obtained for acid-fast bacilli (AFB) staining, mycobacterial culture, and drug susceptibility testing [11]. Tissue specimens should also be examined microscopically, after routine and AFB staining, but the absence of AFB and of granulomas or even failure to culture *M. tuberculosis* does not exclude the diagnosis of tuberculosis. Bacteriological evaluation of the response to treatment in extrapulmonary tuberculosis is often limited by the difficulty in obtaining follow-up specimens. Thus, response often must be judged on the basis of clinical and radiographic findings. Primary tuberculosis (TB) of the thyroid gland is extremely unusual and may have a number of different clinical manifestations. It usually mimics more common conditions such as thyroid adenoma or carcinoma, lymphoma, infective or granulomatous thyroiditis, Graves disease, multinodular goiter, or bacterial abscess. This delays diagnosis, especially if there is no evidence of other organ involvement [12].

Tuberculosis of the thyroid gland is an uncommon disease, and primary involvement of thyroid is even more rare. It is a rare disease even in countries in which tuberculosis is endemic. The diagnosis is often difficult as the clinical presentation has no distinct characteristics. The clinical course of the disease may resemble toxic goiter or acute thyroiditis or may follow a subacute or chronic growth pattern without specific symptomatology. Histologically presence of necrotizing epithelioid cell granulomas along with Langhans-type giant cells are the hallmark of thyroid tuberculosis. Demonstration of acid-fast bacilli by Ziehl−Neelsen (ZN) staining confirms the diagnosis, but this stain is frequently negative in tissue sections [13]. Coming to our case, the patient had low-grade intermittent fever, unquantified weight loss, and night sweating, but she was did not have a cough. Repeated smears from thyroid abscess collection showed extensive caseous necrosis on a dirty background, and GeneXpert of the pus sample was positive. In general, first-line treatment of tuberculosis consists of combination therapy with isoniazid, rifampin (rifapentine and rifabutin under specific situations), pyrazinamide, and ethambutol. In the setting of drug resistance or intolerance to first-line agents, second-line agents may be used (Table 1). Adults with pulmonary or extrapulmonary TB are eligible for the 6-month 2HRZE/4HR regimen, except for those with TB of the central nervous system, bone, or joint, for which some expert groups suggest longer therapy (that is, 9–12 months) [14]. Our patient was also started on 2HRZE/4HR regimen.

**Table 1:** Categories of antituberculosis drugs

Group one: first-line oral antituberculosis drugs (use all possible drugs)
Isoniazid
Rifampin
Ethambutol
Pyrazinamide
Group two: fluoroquinolones (use only one, because they share genetic targets)
Levofloxacin
Moxifloxacin
Gatifloxacin
Ofloxacin
Group three: injectable antituberculosis drugs (use only one, because they share very similar genetic targets)
Capreomycin
Kanamycin
Amikacin
Streptomycin
Group four: less-effective second-line antituberculosis drugs (use all possible drugs if necessary)
Ethionamide/prothionamide
Cycloserine/terizidone
Aminosalicylic acid (acid salt)
Group five: less-effective drugs or drugs on which clinical data are sparse (use all necessary drugs if there are less than four from the other groups)
Bedaquiline
Clofazimine
Amoxicillin with clavulanate
Linezolid
Imipenem–cilastatin
Clarithromycin
Thioacetazone

The decision to initiate combination antituberculosis chemotherapy should be based on epidemiologic information; clinical, pathological, and radiographic findings; and the results of microscopic examination of acid-fast bacilli-stained sputum (smears) (as well as other appropriately collected diagnostic specimens) and cultures for mycobacteria. A purified protein derivative (PPD)-tuberculin skin test may be done at the time of initial evaluation, but a negative PPD-tuberculin skin test does not exclude the diagnosis of active tuberculosis. However, a positive PPD-tuberculin skin test supports the diagnosis of culture-negative pulmonary tuberculosis, as well as latent tuberculosis infection in persons with stable abnormal chest radiographs consistent with inactive tuberculosis. If the suspicion of tuberculosis is high or the patient is seriously ill with a disorder, either pulmonary or extrapulmonary, that is thought possibly to be tuberculosis, combination chemotherapy using one of the recommended regimens should be initiated promptly, often before AFB smear results are known and usually before mycobacterial culture results have been obtained. A positive AFB smear provides strong inferential evidence for the diagnosis of tuberculosis. If the diagnosis is confirmed by isolation of *M. tuberculosis* or a positive nucleic acid amplification test, treatment can be continued to complete a standard course of therapy [15].

When the initial AFB smears and cultures are negative, a diagnosis other than tuberculosis should be considered and appropriate evaluations undertaken. If no other diagnosis is established and the PPD-tuberculin skin test is positive (in this circumstance, a reaction of 5 mm or greater induration is considered positive), empirical combination chemotherapy should be initiated. If there is a clinical or radiographic response within 2 months of initiation of therapy and no other diagnosis has been established, a diagnosis of culture-negative pulmonary tuberculosis can be made and treatment continued with an additional 2 months of INH and RIF to complete a total of 4 months of treatment, an adequate regimen for culture-negative pulmonary tuberculosis. If there is no clinical or radiographic response by 2 months, treatment can be stopped and other diagnoses including inactive tuberculosis considered.

If AFB smears are negative and suspicion for active tuberculosis is low, treatment can be deferred until the results of mycobacterial cultures are known and a comparison chest radiograph is available (usually within 2 months). In low-suspicion patients not initially being treated, if cultures are negative, the PPD-tuberculin skin test is positive (5 mm or greater induration), and the chest radiograph is unchanged after 2 months, one of the three regimens recommended for the treatment of latent tuberculosis infection could be used. These include (1) INH for a total of 9 months, (2) RIF with or without INH for a total of 4 months, or (3) RIF and PZA for a total of 2 months. Because of reports of an increased rate of hepatotoxicity with the RIF–PZA regimen, it should be reserved for patients who are not likely to complete a longer course of treatment, can be monitored closely, and do not have contraindications to the use of this regimen. Patients suspected of having tuberculosis should have appropriate specimens collected for microscopic examination and mycobacterial culture. When the lung is the site of disease, three sputum specimens should be obtained. Sputum induction with hypertonic saline may be necessary to obtain specimens, and bronchoscopy (both performed under appropriate infection control measures) may be considered for patients who are unable to produce sputum, depending on the clinical circumstances. Susceptibility testing for INH, RIF, and EMB should be performed on a positive initial culture, regardless of the source of the specimen [15].

Second-line drug susceptibility testing should be done only in reference laboratories and be limited to specimens from patients who have had prior therapy, who are contacts of patients with drug-resistant tuberculosis, who have demonstrated resistance to rifampin or to other first-line drugs, or who have positive cultures after more than 3 months of treatment. It is recommended that all patients with tuberculosis have counseling and testing for HIV infection, at least by the time treatment is initiated, if not earlier. For patients with HIV infection, a CD4^+^ lymphocyte count should be obtained. Patients with risk factors for hepatitis B or C viruses (for example, injection drug use, foreign birth in Asia or Africa, or HIV infection) should have serologic tests for these viruses. For all adult patients, baseline measurements of serum amino transferases (aspartate aminotransferase [AST], alanine aminotransferase [ALT]), bilirubin, alkaline phosphatase, and serum creatinine and a platelet count should be obtained. Testing of visual acuity and red−green color discrimination should be obtained when EMB is to be used [15]. Coming to our case, her RVI status was nonreactive and all the baseline investigations were in the normal range. The second-line antituberculosis drugs are so classified because of relative lack of clinical data, unfavorable or poorly characterized pharmacokinetic profile, and/or increased incidence and severity of adverse events (Table 2) [16, 17]. Experience with some of these agents is increasing based upon the need for alternative therapies for treatment of drug-resistant TB. The WHO suggests the use of the 6-month treatment regimen composed of bedaquiline, pretomanid, linezolid (600 mg), and moxifloxacin (BPaLM) rather than 9-month or longer (18-month) regimens in MDR/RR-TB patients [18].

**Table 2:** Dosing of second-line antituberculosis drugs in adults

Drug	Daily adult dosage^a^ (normal renal function)	Main adverse effects	Pregnancy
Levofloxacin	1000 mg orally or IV^b^	GI toxicity, CNS effects, rash, dysglycemia, tendonitis or tendon rupture, QT prolongation	Potential choice when there are no suitable alternatives
Moxifloxacin	400 mg orally or IV	GI toxicity, CNS effects, rash, tendonitis or tendon rupture, QT prolongation, dysglycemia	Potential choice when there are no suitable alternatives
Capreomycin^c^	15 mg/kg IM or IV (max 1 g)	Auditory and vestibular toxicity, nephrotoxicity, electrolyte disturbances	Avoid
Kanamycin^c,d^	15 mg/kg IM or IV (max 1 g)	Ototoxicity, nephrotoxicity	Avoid
Amikacin^c,d^	15 mg/kg IM or IV (max 1 g)	Ototoxicity, nephrotoxicity	Avoid
Streptomycin^c^	15 mg/kg IM or IV (max 1 g)	Vestibular and ototoxicity, neurotoxicity, nephrotoxicity	Avoid
Ethionamide	15 to 20 mg/kg orally as a single daily dose or 2 divided doses (max 500 mg twice daily)	GI and hepatic toxicity, neurotoxicity, hypothyroidism, optic neuritis Pyridoxine 50 to 100 mg orally per day may be useful in preventing or reducing neurotoxicity	Potential choice when there are no suitable alternatives
Cycloserine	10 to 15 mg/kg orally in 2 divided doses (max 500 mg twice daily)	Psychiatric symptoms, headaches, seizures Pyridoxine 50 mg (oral once per day) for every 250 mg of cycloserine may be useful in preventing or reducing neurotoxicity	Potential choice when there are no suitable alternatives
Para-aminosalicylic acid	8 to 12 g orally in 2 or 3 divided doses	GI toxicity, malabsorption, hypersensitivity, hepatitis, hypothyroidism	Potential choice when there are no suitable alternatives

*IM* intramuscular, *IV* intravenous, *GI* gastrointestinal, *CNS* central nervous system, *max* maximum

^a^Dosage may need to be adjusted for renal impairment

^b^Greater efficacy has been observed with administration of 1000 mg (compared with 500 mg) for treatment of multidrug-resistant tuberculosis

^c^Generally given 5−7 times per week (15 mg/kg, or a maximum of 1 g per dose) for an initial 2−4 months, and then (if needed) 2 or 3 times per week (20−30 mg/kg, or a maximum of 1.5 g per dose). For patients > 59 years old, dosage is reduced to 10 mg/kg (maximum 750 mg per dose). Dosage should be decreased if renal function is diminished

^d^For patients who are overweight or obese, dose is based on ideal body weight or dosing weight. Adjust dose based on serum concentration monitoring for target trough < 1 mcg/mL and target peak of 56−64 mcg/mL for once daily administration

The significance of anterior neck swelling lies in the fact that the majority of cases remain asymptomatic, thus patients do not seek medical advice until much later. Thus, they remain undiagnosed and serve as a potential source of morbidity and mortality in the community. On the other hand, the diagnosis is often overlooked in view of other common causes of anterior neck swelling, such as multinodular colloid goiter or thyroid neoplasms. In our patient, who reported from endemic area of tuberculosis, the anterior neck swelling, in the absence of other significant clinical and laboratory findings, initially misled the clinician. Lack of suspicion of tuberculosis led to a long period of suffering for the patient. The patient was very satisfied with the intervention and care given.

## Conclusion

We present the case of a 60-year-old Ethiopian female patient diagnosed to have thyroid tuberculosis mimicking multinodular goiter through cytopathological examination and GeneXpert and was started on 2 RHZE/4 RH 3 tablets by mouth per day and pyridoxine 50 mg by mouth/day for 6 months after she presented with multinodular anterior neck swelling. Anterior neck swelling due to infection with *Mycobacterium tuberculosis* needs to be included in the spectrum of unusual presentations of tuberculous infections, and tuberculosis as a differential diagnosis needs to be kept in mind when a patient with anterior neck swelling is encountered from an area endemic for tuberculosis. We believe that, with further accumulation of cases and experience in the future, our understanding of primary thyroid tuberculosis will become enhanced, and the diagnosis and treatment of this disease will be improved.

## Data Availability

Data sharing does not apply to this article as no new data were created or analyzed in this study.

## Abbreviations

TB: Tuberculosis
Anti-tb: Anti-tuberculosis
RHZE: Rifampicin (R), ethambutol (E), isoniazid (H), pyrazinamide (Z)
RH: Rifampicin (R), isoniazid (H)
BPaLM: Bedaquiline, pretomanid, linezolid, and moxifloxacin
MDR/RR-TB: Multidrug-resistant/rifampicin-resistant tuberculosis
EPTB: Extrapulmonary tuberculosis
LTBI: Latent tuberculosis infection
RVI: Retroviral infection
